# Medical education in Iraq: issues and challenges

**DOI:** 10.5116/ijme.58b1.c927

**Published:** 2017-03-11

**Authors:** Mustafa Al-Shamsi

**Affiliations:** 1 Department of Public Health, Ministry of Health, Iraq

**Keywords:** Medical education, historical perspective, issues and challenges, Middle East, Iraq

## Introduction

Medical education in Iraq was one of the best educational systems in the region, especially during the 1980s. Following this golden period, the educational system suffered from regression and descent in both the academic curriculums that are taught and teaching techniques of both undergraduate and postgraduate physicians. This was due to multiple factors: including the wars, sectarian conflicts, and financial sanctions which had a lasting effect on medical education and training. Today, medical education and training in Iraq face multiple challenges resulting from a lack of facilities, financial support, and doctors' emigration due to violence and political unrest.^1^^,^^2^ there are a few articles published on the medical education in Iraq. The purpose of this article is to give the international readers an overview of the medical education in Iraq. Emphasizing the current challenges that are faced by the educational system and providing some points for improvements.

### Historical perspective of medical education in Iraq

The first medical college in Iraq was the Iraqi Royal Medical College (now Baghdad College of Medicine) founded in 1927 by a group of Iraqi doctors who graduated from international medical schools. Harry Sanderson (a British Physician) was the first dean of medicine in Iraq. He established the first medical curriculum in coordination with the Royal College of Surgeons (Edinburgh) and set a strategic plan for medical education in Iraq.^3^ In the subsequent years, the Royal Medical College developed and presented itself as one of the best medical schools in the Arab world in terms of academic teaching and research.

Medical schools continued to expand in the rest of Iraq to involve foundations of Mousel, Basra, and Mustansiriya medical colleges. The medical training and education system reached its peak during the 1980s. Iraqi medical schools became a destination for scholars from the Middle East and Africa. The quality of medical training was in accordance with international guidelines and it was continually updated. Iraqi doctors had been sent on a regular basis, through fellowships, to receive postgraduate training. These efforts improved the health care system in various aspects. For example, the Medical City Hospital was one of the largest and best equipped hospitals in the Middle East during 1980s. Additionally, the first heart center in the Arab world was in Iraq.

In 1988 the educational system, as a whole, had been affected by the Iraqi-Iranian War. After economic sanctions in the 1990s, there was a further burden on the educational system. The financial crisis had a huge impact on medical education and training. The government seized the fellowship training programs. As a result, the medical education system suffered from a devastating lack of resources and development.^4^ The government regime prohibited doctors from traveling for training abroad even if they paid for their own training. The regime also banned the academic transcripts for medical graduates, a tactic that intends to prevent the physicians from leaving Iraq.

The conflict and political unrest that followed 2003 further burdened medical education. The frequent threats and assaults that accompanied the civil order decline causing an exodus of the majority of senior professors from Iraq.^5^^-^^7^ This compulsory migration of medical workforce and professional competencies had a huge adverse effect on the system leadership, training, and the medical education in Iraq.

### Orientation of the undergraduate and post-graduate medical education in Iraq

Currently, there are twenty-four accredited medical colleges in Iraq.^8^ All the colleges are governmental, and operate under the patronage of the Iraqi Ministry of Higher Education. The undergraduate medical education is based on a six year British curriculum. It’s free; all medical students have access to lectures, textbooks, and clinical resources without paying a tuition fee. The curriculum is taught completely in English. All the colleges follow the traditional mode of teaching; which includes traditional lectures, basic science laboratories, and clinical based teaching. Tikrit University College of Medicine (TUCOM) is the only college in Iraq that introduced a block mode of teaching. In the block mode, the students are encouraged to engage in group discussions that include self-teaching.^9^ It is similar to problem-based learning (PBL), yet, it still lacks a lot of elements of a typical problem-based learning technique. The mode of assessment consists of written examinations in the first three basic science years, and written combined with an oral assessment in the last three clinically oriented years. The final assessment degree is 100%. The criteria for passing each grade require the student to score at least 50%.

Upon graduation, the physicians go through a year of clinical clerkship in the major branches of medicine. During the second year, all physicians must serve in the rural and remote cities before they are allowed to pursue their postgraduate studies. After two years of general training, the physicians then have the right to pursue specialized postgraduate training. The physicians can choose to pursue a medical specialization in either the Iraqi or the Arabian board of medical specialty. Generally speaking, the residency program is four years for the medical specialty and five years for the surgical specialty.

### The current situation of medical education in Iraq: issues and challenges

Currently, the medical education in Iraq faces multiple challenges that are represented by many factors including, but not limited to the old curriculum, traditional teaching methods, and unavailability of the proper facilities. Colleges are concentrating more on students’ attendance and less on updating the curriculum-which is outdated. Each medical college has its own curriculum, and there are no guidelines that apply to all medical schools. Learning is still traditional, and characterized by a teacher-centered approach. This method centers on the teacher who is controlling the course content and the methods of presentation, i.e. focusing on teaching rather than learning. The modern style of medical education has not yet been introduced: the one which depends on a student-centered approach in which the students are encouraged to take greater responsibility for learning decisions and to question what and how they learn; while they are supervised by a mentor (PBL).^10^

One of the challenges of medical education in Iraq is providing appropriate clinical exposure to students and trainees in different phases of their careers. This is because of the lack of adequate clinical expertise in addition to a limited number of teaching centers and facilities. The students, due to this constraint find it difficult to learn. They fail to apply the theoretical knowledge that they previously learned. This leads the students to deteriorate quickly after first being interested in studying medicine i.e. they lose the motivation they brought when they applied to study medicine.

Post-graduate training is a different dilemma. While undergraduates receive poor teaching, postgraduates complain of not receiving any teaching at all. Some trainees feel disappointed, stressed, and unsupported due to the absence of proper systemic supervision during the residency period. This makes the feedback they receive more destructive than constructive. On their way toward career specialization, they may face politicization of both the health and education systems. This makes it impossible to get the specialty that the physician is pursued without having connections as the medical specialization commonly decided by who you know rather than your qualifications. They may also face humiliation from their supervisors, which causes the majority of them to leave their careers. Furthermore, being a trainee in a hostile environment where there are frequent threats and both verbal and physical assaults due to the security concern makes the mission more difficult.^11^^,^^12^

## Conclusions

The situation of medical education in Iraq is complex and determined by many factors, including politics, financial matter, planning, and security situation. At present, the strategic plan to shape the future of medical education in Iraq is vague. Until now, no real steps have been taken toward improving the educational system. The educational system may need to be revised and reformed again by several ways. For example, the government may launch a fellowship programs to send physicians for training abroad in order to rebuild the curriculum of medical education in Iraq according to international guidelines. Moreover, the solutions of medical education issues are not only lying in the syllabus alone, but more in the teaching methods. Both trainees and medical students should have an educational supervisor that helps in both the personal and the professional development. The role of supervision should not only be limited to the clinical duties. Teachers need to be familiar with their role as a facilitator of students and learn to guide rather than control the students i.e. applying the student-center method. At the same time, the trainees should encourage to take more responsibility toward self-teaching. In other words, active learning should be fortified by a modern method of teaching such as PBL. Technology usage in Iraqi universities in general is still poor and needs to be developed. Introduction of the online teaching would be beneficial for the students. For instance, online courses such as the massive online course teaching (MOOC), and interactive teaching sessions that connect them with their peers abroad.

### Acknowledgements

The author would like to thank Dr Stephanie Waggle and Ms Rebecca Wadness for editing and proofreading this manuscript.

### Conflicts of Interest

The author declare**s** that they have no conflict of interest.
